# Biopolymer-Capped Pyrazinamide-Loaded Colloidosomes: *In Vitro* Characterization and Bioavailability Studies

**DOI:** 10.1021/acsomega.3c03135

**Published:** 2023-07-07

**Authors:** Avi Singh, Sabya Sachi Das, Janne Ruokolainen, Kavindra Kumar Kesari, Sandeep Kumar Singh

**Affiliations:** † Department of Pharmaceutical Sciences and Technology, Birla Institute of Technology, Mesra, Ranchi 835215, Jharkhand, India; ‡ School of Pharmaceutical and Population Health Informatics, DIT University, Dehradun 248009, Uttarakhand, India; § Department of Applied Physics, School of Science, Aalto University, Espoo 00076, Finland; ∥ Faculty of Biological and Environmental Sciences, University of Helsinki, Viikinkaari 1, 00790 Helsinki, Finland

## Abstract

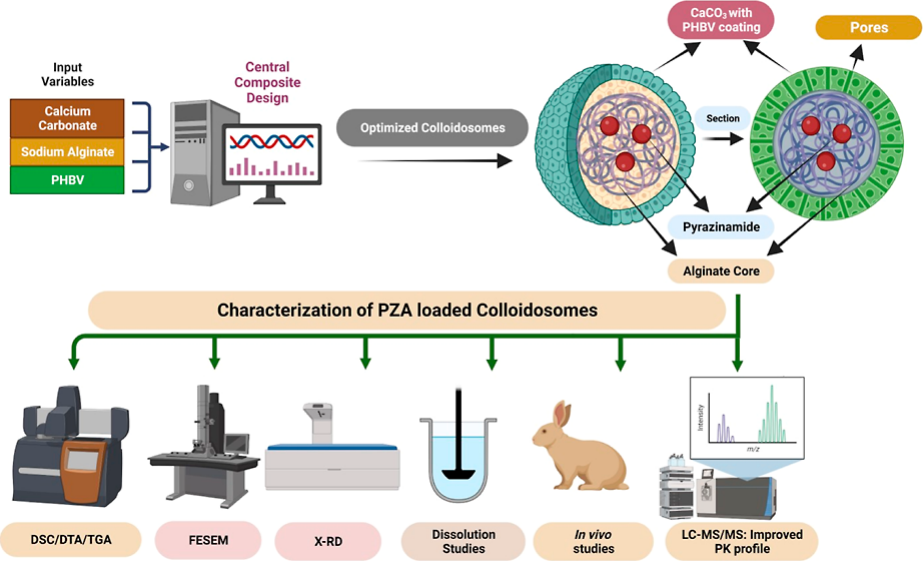

This study aimed to prepare colloidosome particles loaded with pyrazinamide (PZA). These drug-loaded colloidosomes were prepared using an *in situ* gelation technique using a central composite design with a shell made of calcium carbonate (CaCO_3_) particles. Optimal amounts of 150 mg of CaCO_3_, sodium alginate (2%), and 400 mg of poly(3-hydroxybutyrate-*co*-3-hydroxy valerate) (PHBV) concentration resulted in the maximum drug loading and efficient release profile. Field emission scanning electron microscopy results showed spherical porous particles with a good coating of the PHBV polymer. Additionally, Fourier transform infrared (FTIR) spectroscopy, differential scanning calorimetry (DSC), thermogravimetric and differential thermal analysis (TGA-DTA), and X-ray diffraction (XRD) analysis showed good compatibility between the drug and excipients. The pharmacokinetic studies demonstrated that the drug-loaded colloidosomes resulted in 4.26 times higher plasma drug concentrations with *C*_max_ values of 32.386 ± 2.744 mcg/mL (PZA solution) and 115.868 ± 53.581 mcg/mL (PZA-loaded colloidosomes) and AUC_0–*t*_ values of 61.24 mcg-h/mL (PZA solution) and 260.9 mcg-h/mL (PZA-loaded colloidosomes), indicating that colloidosomes have the potential to be effective drug carriers for delivering PZA to the target site.

## 1 Introduction

Tuberculosis (TB) is a prevalent and contagious disease caused by the spread of different strains of Mycobacterium, commonly *Mycobacterium tuberculosis* and *Mycobacterium bovis*. It primarily affects the lungs but can also impact other organs.^1^ Despite the availability of treatment options for over half a century, TB remains one of the leading causes of preventable deaths worldwide. A recent report shows that the total number of incident TB patients in India (new and relapse) notified in 2021 was 19,33,381, which is 19% higher than in 2020 (16,28,161).^2^

Pyrazinamide (PZA) is a first-line medication used to treat active TB.^3^ It is an FDA-approved antibacterial drug essential to multiple drug therapy. PZA is water-soluble and has a 15 mg/mL solubility at 25 °C.^3,4^ It is only active at a slightly acidic pH and is typically administered daily with rifampicin and isoniazid during the initial 2 months of TB treatment.^5^ The drug is well-absorbed and distributed throughout the body. In recent decades, researchers have developed nanocarriers such as stealth liposomes and poly(d-l-lactide-*co*-glycolide) microspheres to improve the efficacy of chemotherapy against TB.^6,7^ However, poor patient compliance remains a significant concern, leading to the necessity of an efficient therapeutic system for targeting various microbial infections with better therapeutic effects.^8,9^ Dry powder inhalers are a viable option as they can efficiently deliver large amounts of drugs to the lungs.^10^ However, a recent study showed that the PZA detected in alveolar macrophages after inhalation of dry PZA powder was moderately low due to its premature degradation.^7^ The scientific community’s acceptance of nanotechnology-based drug delivery systems opens up current and future research opportunities.^11−14^ These technologies may serve as a viable option for treating chronic diseases such as TB as they can cross biological barriers and target specific sites such as the cellular reservoirs of *M. tuberculosis*.^12^

Colloidosomes are a type of vesicular drug delivery system that has high encapsulation efficiency and permeability.^15^ They are small, hollow, and elastic capsules made of a layer of colloidal particles at the interface of an emulsion droplet.^15,16^ These particles self-assemble to form the shell, which has interstitial pores that control the release of encapsulated materials and permeability.^17^ In this study, we attempted to use calcium carbonate (CaCO_3_) particles as the shell for colloidosomes loaded with pyrazinamide (PZA) as a way to control the release of the drug. The self-assembled CaCO_3_ layer around the emulsion droplet acts as a stabilizer and slows down the drug’s release, demonstrating the potential of colloidosomes as a controlled drug delivery system.^18^

d-Glucono δ-lactone (GDL) in the aqueous phase can decrease the pH and aid in releasing calcium ions (Ca^2+^), which serve as cross-linkers. These particles can be manipulated by adjusting their size, shape, and arrangement around the liquid droplet.^19^ When arranged in a consistent pattern, these CaCO_3_ particles form shells around the liquid emulsion droplets, called colloidosomes.^17^ Colloidosomes have a variety of applications in drug delivery systems and as carriers for various substances such as enzymes, proteins, food additives, fragrances, and flavors, among others.^15,20^ The size of the colloidosome particles, mechanical strength, and permeability are key characteristics that affect the release of substances.^16,20^

PZA, a weak acid, is more effective against *M. tuberculosis* at lower pH levels according to the Henderson–Hasselbalch equation.^21^ This was further confirmed by the study in which the activity of PZA was amplified in the presence of certain weak acids.^21^ A study found that pH 3 and 5 were particularly effective environments for PZA.^22^ It is believed that a low pH is necessary for PZA to enter and attack the mycobacteria. Some theories suggest that a low pH causes the protonation of extracellular pyrazinoic acid, which is necessary for PZA to enter the mycobacteria and have its antimicrobial effect. The reduced membrane potential at low pH may also allow PZA to deplete the mycobacteria’s energy.^23^ Based on the previous discussion, it is also important to have an acidic environment around PZA in the dosage form. This was achieved in the current study by incorporating GDL into the formulation, which helps create an acidic environment and aids in cross-linking by releasing Ca^2+^.

The main goal of this work is to optimize and create a colloidosome drug delivery system using a central composite design (CCD) with three factors and five levels, which required 16 experimental runs. The drug loading (*Y*1) and percent release (*Y*2) of each prepared formulation were evaluated. Additionally, the study aims to evaluate the pharmacokinetics and *in vivo* effects of PZA-loaded colloidosomes using GastroPlus software.

## 2 Materials and Methods

### 2.1 Materials

PZA was obtained from Thermo Fischer, Corn Oil from Fluka Biochemicals, sodium alginate, poly(3-hydroxybutyrate-*co*-3-hydroxy valerate) (PHBV) polymer, and GDL from Sigma-Aldrich. Escitalopram (internal standard) was purchased from Sigma-Aldrich (India). Polypropylene tubes, pipette tips, and measuring tubes were obtained from Tarsons (India). Tertiary butyl methyl ether was purchased from Merck (India). HPLC-grade formic acid and water were obtained from Rankem (India) and HPLC-grade methanol from Fischer Scientific. The analytical column used was Reprosil Gold from Dr. Maison GmBH. All aqueous solutions were prepared using Milli Q/Elix water from Millipore. All other chemicals used were of analytical grade.

### 2.2 Methods

#### 2.2.1 Preparation of PZA-Loaded Colloidosomes

After evaluating various preparation methods, we chose the *in situ* gelation technique due to its benefits, such as structural integrity and stability.^24^ The critical formulation variables that have a significant impact on the percentage of drug loading (*Y*1) and drug release (*Y*2) were determined using CCD (Design-Expert 13.0.3.0 software, Stat-Ease, Inc., USA). PHBV (400 mg) was dissolved in chloroform (2.0 mL) and added to the oil phase, which consisted of previously dispersed CaCO_3_ microparticles in corn oil. The aqueous phase was made up of sodium alginate and GDL. The aqueous phase was added to the oil phase drop-wise with continuous stirring to create a water/oil (w/o) emulsion. After a set time, the emulsion was centrifuged at 5000 rpm to remove the oil phase and washed three times with water to ensure the complete removal of the oil phase.

The independent variables selected for the study were the amounts of CaCO_3_, sodium alginate, and PHBV, designated as A, B, and C, respectively. The amounts of CaCO_3_, sodium alginate, and PHBV ranged from 49.43 to 175.57 mg, 0.977 to 6.02%, and 131.82 to 468.18 mg, as shown in Table 1.

**Table 1:** Dependent and Independent Variables with Their Coded and Actual Values

levels								
	low		middle		high		–α	+α
independent variables	coded	actual	coded	actual	coded	actual		
A: CaCO_3_ (mg)	–1	75.00	0	112.50	+1	150.00	49.43	175.57
B: sodium alginate (%)	–1	2.00	0	3.50	+1	5.00	0.977	6.02
C: PHBV (mg)	–1	200.00	0	300.00	+1	400.00	131.82	468.18

dependent variables	low	high
*Y*1: drug loading (%)	18.10	88.25
*Y*2: percent release (%)	6.21	21.65

The coded values used were −1 for low, +1 for high, and 0 for intermediate. The star points were designated as +α and −α. A total of 16 runs were designed based on CCD (Table 2).

**Table 2:** Experimental and Predicted Values of Drug Loading (*Y*1) and Percentage Release (*Y*2)

	factors			responses			
				*Y*1 (drug loading)		*Y*2 (percent release)	
S. no	*X*1 CaCO_3_ (mg)	*X*2 sodium alginate (percent)	*X*3: PHBV (mg)	actual	predicted	actual	predicted
1	75.00	5.00	400.00	65.02	64.98	11.92	9.58
2	112.50	6.02	300.00	55.24	53.78	6.22	9.49
3	112.50	0.97	300.00	49.26	55.59	21.57	20.09
4	150.00	2.00	400.00	85.33	80.75	16.86	15.62
5	150.00	5.00	400.00	88.25	89.59	11.88	9.32
6	49.43	3.50	300.00	35.02	35.95	14.17	15.00
7	150.00	5.00	200.00	60.32	57.54	14.55	13.69
8	112.50	3.50	300.00	40.25	40.83	12.30	14.79
9	150.00	2.00	200.00	67.72	64.32	19.16	19.99
10	112.50	3.50	300.00	42.25	40.83	11.78	14.79
11	175.56	3.50	300.00	81.36	85.31	13.58	14.57
12	75.00	2.00	400.00	61.02	60.35	13.77	15.88
13	112.50	3.50	468.17	82.25	82.94	11.77	11.11
14	75.00	2.00	200.00	35.02	30.24	21.66	20.25
15	75.00	5.00	200.00	18.10	19.24	15.87	13.95
16	112.50	3.50	131.82	26.48	30.67	19.53	18.46

Out of the 16 runs, eight were cubic points, two were center points of levels, and six were axial points. The ratio of GDL to CaCO_3_ was kept constant at 1:3. All the formulations were dried and stored for further characterization.

#### 2.2.2 Characterization Studies

##### 2.2.2.1 Fourier Transform Infrared Spectroscopy

The infrared spectrum of pure PZA, individual excipients, and their physical mixtures (PMs)(1:1) was examined to check for any possible interactions between the drug and excipients. The optimized formulation (OF) of colloidosomes was also scanned and analyzed in the 4000–600 cm^–1^ range using the KBr disc/pellet method (Shimadzu FTIR 8400S). Before analysis, samples were mixed with KBr and manually compressed into a pellet to obtain the transmittance report. The calculations were performed using IR Solutions software for background subtraction, baseline correction, normalization, and spectrum recording tasks.^25^

##### 2.2.2.2 Powder X-ray Diffraction Studies

To acquire the diffraction patterns of pure PZA, excipients, and the OF, a powder X-ray diffraction (PXRD) analyzer (Rigaku-Miniflex, Tokyo, Japan) was used. The analyzer features a 30 kV generator, 15 mA anode tube, and Cu-Kα radiation. The scanning range was set at 2θ from 3 to 80° with a step size of 0.02° and a scanning rate of 2° per minute.^25^

##### 2.2.2.3 Thermogravimetric-Differential Thermal Analysis

The thermal degradation of pure PZA, excipients (GDL, sodium alginate, and PHBV), and the OF were examined using thermogravimetric-differential thermal analysis (TG-DTA). The samples pre-weighed for analysis were placed in aluminum pans and gradually heated from room temperature to 800 °C at a heating rate of 10 °C min^–1^. Dry nitrogen was used to purge the samples at a rate of 50 cm^3^ min^–1^ in a calibrated system using a DTG 60 instrument (Shimadzu, Japan). The peak degradation and weight loss were analyzed to determine the thermal stability of the samples.^25^

##### 2.2.2.4 Field Emission Scanning Electron Microscopy

The surface topology of the drug (PZA), various excipients, and the OF were examined using field emission scanning electron microscopy (FESEM) (Carl Zeiss; Sigma 300, Germany) by mounting dried and powdered samples (previously coated with gold in an inert atmosphere) onto brass stubs using double-sided tape. The samples were scanned at 5 kV with different resolutions to understand the surface topology better.^25^

##### 2.2.2.5 *In Vitro* Drug Release Study

The *in vitro* release study for all the formulations (OF-1 to OF-16) and the OF was conducted on a USP dissolution type 2 apparatus at 50 rpm and ambient temperature (37 ± 0.5 °C) in 900 mL of the Millipore water medium. As per the definition mentioned in the European Pharmacopeia, the sink conditions are well-defined as the dissolution medium’s volume, which is at least 3 to 10 times of the saturation volume.^26^ In our study, to maintain sink conditions, 5.0 mL of aliquots were taken out at regular intervals for up to 24 h, and an equal volume of fresh medium was added at each step. Each sample was then analyzed using a UV spectrophotometer (UV 2450, Shimadzu) at a predetermined lambda max (λ_max_) of 268.6 nm. The percent drug release and dissolution efficiency were then calculated.

#### 2.2.3 Pharmacokinetic Studies

Animal studies were conducted on white New Zealand rabbits (1.5–2.5 kg body weight) with approval from the Institutional Animal Ethical Committee (no. 1972/PH/BIT/130/21/IAEC) of the Department of Pharmaceutical Sciences and Technology, Birla Institute of Technology, Mesra, Ranchi. Before the study, rabbits were housed and acclimatized for 2 weeks in standard conditions with access to food and water. The rabbits were made to fast overnight and randomly divided into two groups before experimentation. Group 1 was administered with only PZA solution (free drug) at a dose of 30 mg/kg orally and group 2 with the colloidosome formulation loaded with PZA. Blood samples were systematically collected up to 24 h from the marginal ear vein after oral administration at pre-dose, 0.25, 0.5, 1, 2, 3, 6, 9, 12, and 24 h. The samples were collected in K3-EDTA tubes and centrifuged at 3500 rpm for 30 min at a temperature of 4° C. The corresponding plasma samples were stored at −20 °C until further analysis. The rabbit plasma was then examined using a solid-phase extraction procedure against the internal standard (escitalopram) by liquid chromatography-electrospray ionization mass spectrometry (LC-ESI-MS/MS). The chromatographic separations were achieved on a Reprosil gold analytical column (50 mm × 4.6 mm, 5 μm) (Shimadzu, Japan) using a mobile phase composition of methanol and water acidified with 0.1% formic acid (70:30% v/v) at a flow rate of 0.3 mL per minute and injection volume of 10 μL.

The pharmacokinetic parameters were calculated by the non-compartmental analysis method, which is a more straightforward and efficient analysis method. The PKPlus module of GastroPlus software (version 9.0, Simulations Plus Inc., Lancaster, CA, USA) was used for this purpose. This software is a widely used tool for pharmacokinetic and pharmacodynamic simulations in the pharmaceutical industry. The pharmacokinetic study involved the collection of plasma samples at various time points after the drug administration. These samples were then analyzed to determine the drug concentration in the plasma. The data obtained from these analyses were plotted to generate concentration–time plots. These plots visually represent the drug concentration in the plasma over time. From these plots, several pharmacokinetic parameters were obtained, such as the maximum concentration (*C*_max_), time to reach maximum concentration (*T*_max_), and area under the curve (AUC) from time zero to the last measured concentration (*C*_last_). *C*_max_ represents the highest drug concentration in the plasma, *T*_max_ represents the time when the maximum concentration occurs, and AUC represents the total amount of drug in the body over time. These parameters provide important information about the drug’s pharmacokinetics, such as its absorption, distribution, metabolism, and excretion in the body.

## 3 Results and Discussion

### 3.1 Optimization Studies

We chose the *in situ* gelation technique among the various methods for preparing colloidosomes due to its advantages. We screened different excipients to prepare CaCO_3_ microparticles.^27^ PHBV was selected as a polymer for coating the layer on CaCO_3_ particles. For the aqueous phase, GDL was used in a ratio of 1:3 (GDL/CaCO_3_), and alginate was used for cross-linking. Various formulations (a total of 16) were made according to the CCD design (Table 2). After preparation, the formulations were screened, and the best OF was selected for further study. The effect of each selected variable, mentioned above, over the responses that are crucial for attaining the OFs, is elaborately discussed and systematized in the following sections.

#### 3.1.1 Drug Entrapment/Loading

Optimizing drug loading is crucial for various factors that influence the responses. It is an essential factor in preparing nano drug carrier systems as it significantly affects the final product, such as its efficacy, productivity, robustness, and cost.^28,29^ It was observed from the levels of significance of regression coefficients that only CaCO_3_ concentration (CaCO_3_) (*X*1) and PHBV concentration (*X*3) had a significant contribution to the regression model. The reduced coded equation obtained for the response *Y*1 was
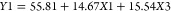
1

**Figure 1:**
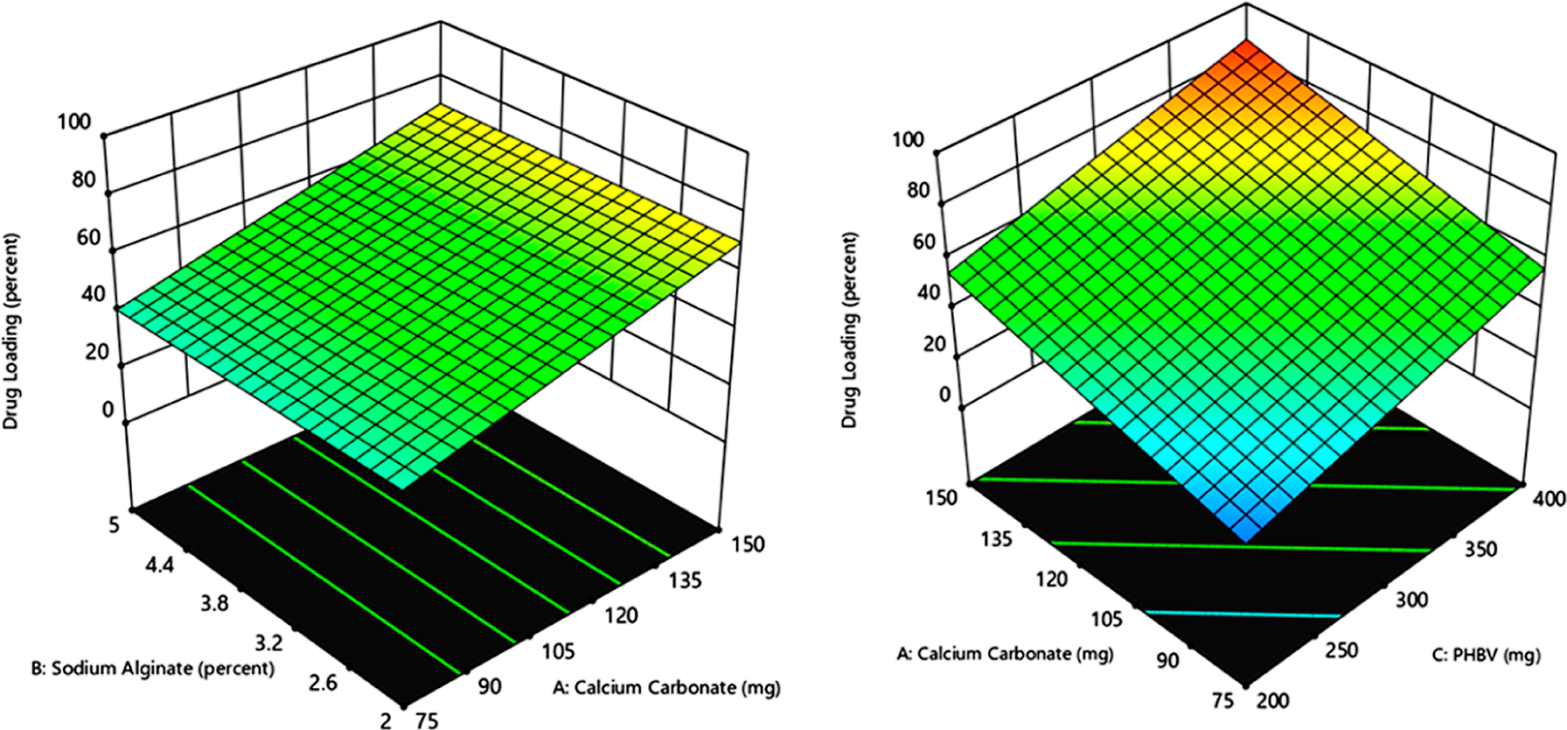
3-D Response surface plots showing the effect of CaCO_3_, sodium alginate, and PHBV on the percent drug loading.

The ANOVA of the given model equations indicates a good fit to the responses, supported by the *F*-value of 43.77 (*p* < 0.0001) with a comparable predicted *r*^2^ (0.8225) and adjusted *r*^2^ (0.8508). The response (*Y*1) for the formulations OF-1 to OF-16 ranged from 18.10 to 88.25% (Table 2). It was also observed that an increase in *X*1 (CaCO_3_) and *X*3 (PHBV) leads to an increase in the drug loading parameter. Contour lines ran almost parallel to one another, thus ruling out any potential interactions between these factor levels (Figure 1). Therefore, it can be inferred that higher concentrations of CaCO_3_ and PHBV should be used to maximize drug loading.

#### 3.1.2 *In Vitro* Release Studies

*In vitro*, release study is a crucial parameter for understanding the systemic absorption of a drug and analyzing the release mechanism in the dissolution medium. The studies were performed under physiological conditions of 37 ± 0.5 °C, which can help predict *in vivo* performance.^30,31^ The concentration of sodium alginate and the amount of PHBV significantly influenced the response *Y*2. The coded equation generated for the response *Y*2 was
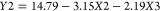
2

**Figure 2:**
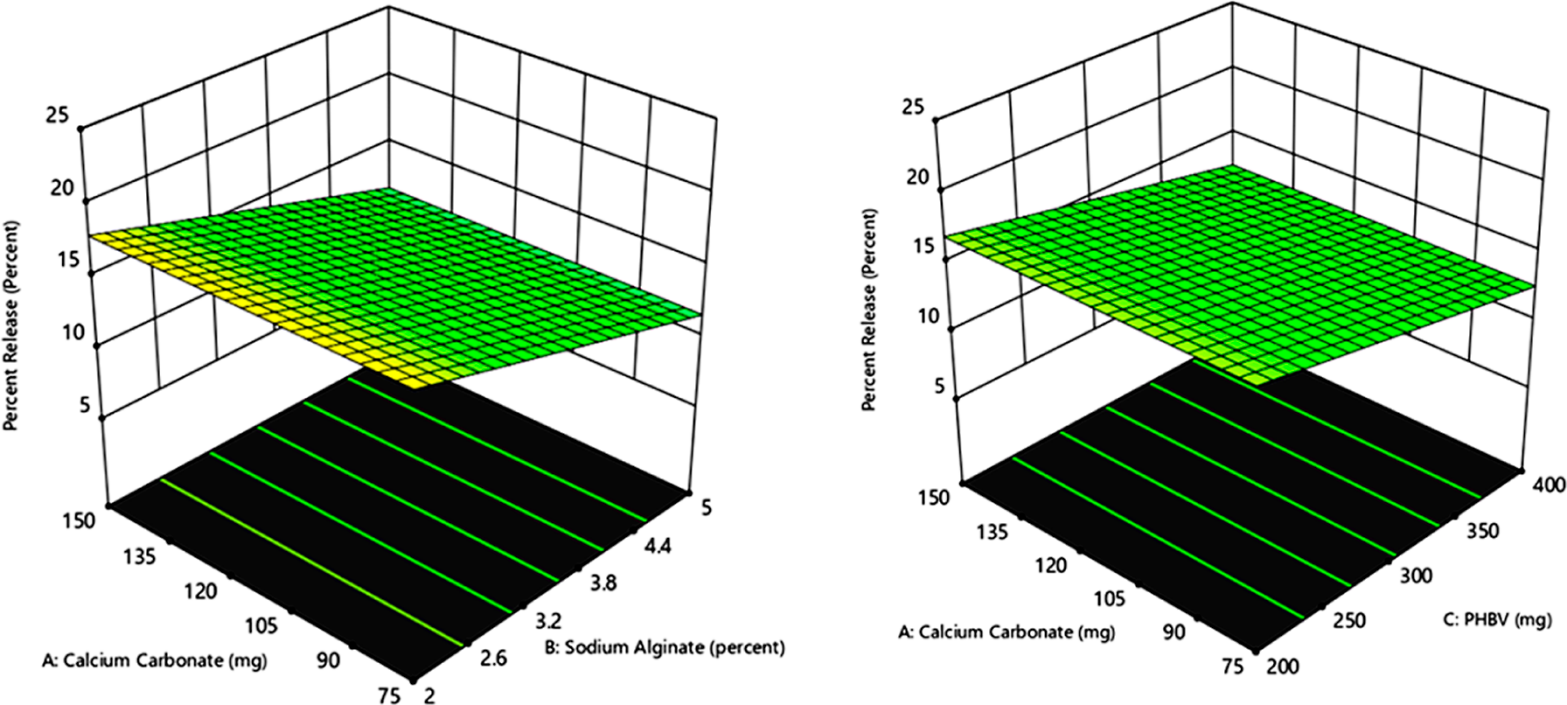
3-D Response surface plots showing the effect of sodium alginate, CaCO_3_, and PHBV on percent release.

The coded equation showed that the predicted *r*^2^ (0.6631) is in close agreement with the adjusted *r*^2^ (0.7460). The ANOVA analysis suggests that the estimated response is well described by the selected model, which was further supported by the *F*-value of 23.03 (*p* < 0.0001). Figure 2 also shows a linear decreasing trend of the percentage release with sodium alginate (*X*2) concentration and PHBV (*X*3).

The interaction plot between the response *Y*2 and factor levels showed linear and parallel lines, indicating no interactions between *X*2 and *X*3. It can be inferred that low amounts of PHBV and alginate need to be used to optimize and maximize percent release. This further indicates that the release of PZA was significantly controlled when higher alginate and PHBV were used in formulations.

### 3.2 Identification and Evaluation of the OF Using the Desirability Function

**Figure 3:**
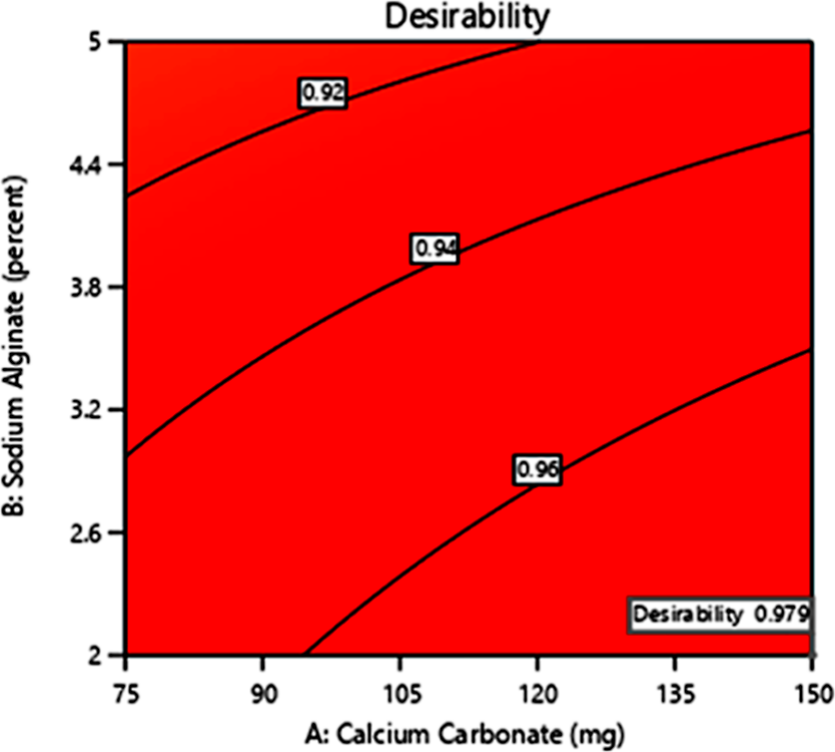
Response surface plot of overall global desirability of the OF (0.979).

The primary goal of optimizing a pharmaceutical formulation is to identify the best-desired formulation with high-quality and robust characteristics at the level of factors from which it can be made. After obtaining the coded equations of both responses (*Y*1 and *Y*2), the process was optimized for the selected responses. To do this, a numerical technique known as the desirability function was used to obtain the desired response using the Design Expert software.^32,33^ The desired responses are assigned by depicting a goal of variables to either minimize, maximize, or fall within a range under the given set of constraints. The correlation between the independent variables and the responses was seen by plotting the desirability response surface curve. The constraints of the independent factors kept in the study were CaCO_3_ concentration *X*1 (in the range of 49.43–175.56 mg), sodium alginate concentration *X*2 (in the range of 0.97–6.02 mg), and the amount of PHBV polymer *X*3 (in the range of 131.82–468.17 mg) to maximize *Y*1 and *Y*2, *i.e.*, drug loading and percent release. The combined global desirability factor was found to be 0.979 (Figure 3).

To confirm and validate the optimization process, a new colloidosome formulation (OF) was prepared using the predicted factors, which consist of 150 mg of CaCO_3_, 2% of sodium alginate, and 400 mg of PHBV polymer, and evaluated for the responses. The response of the OF was as follows: a drug loading of 85.33% and a percent release of 16.86%. The predicted values of the responses are listed in Table 3.

**Table 3:** Validation of Responses Drug Loading (*Y*1) and Percent Release (*Y*2) Using CCD

factors			
	CaCO_3_ conc. (mg) (*X*1)	alginate amount (%) (*X*2)	PHBV conc. (mg) (*X*3)
responses	predicted value	observed value	% biased
*Y*1	80.75	85.33	–5.37
*Y*2	15.62	16.86	–7.35

The percentage biases of the OF were found to be −5.37 and −7.35% for the responses, indicating a good correlation between the predicted and observed values (Table 3). This illustrates the reliability of the optimization process in predicting the OF.

### 3.3 Characterization Studies

#### 3.3.1 Fourier Transform Infrared Spectroscopy

**Figure 4:**
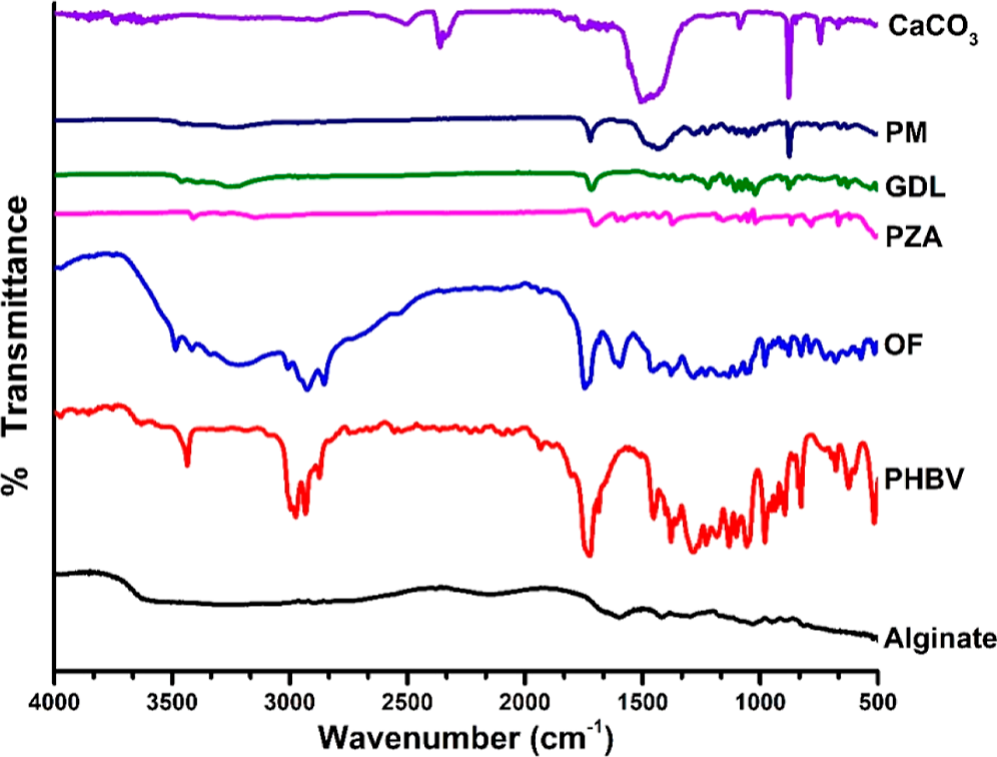
FTIR spectra of alginate, PHBV, OF, PZA, GDL, PM (1:1), and CaCO_3_.

The drug’s and excipients’ compatibilities were checked by the infrared profile of the pure drug as interactions may lead to remarkable changes. IR spectra of PZA, individual excipients (alginate, GDL, PHBV, and CaCO_3_), and PM of the drug with individual excipients are given in Figure 4.

PZA showed characteristic absorptions at 3409 cm^–1^ due to N–H stretching, 3288 cm^–1^ (*sym* N–H), 3145 cm^–1^ due to stretch of the C–H band and carboxylic O–H group, 3150 cm^–1^ (C–H stretch), and 1705 cm^–1^ due to the carbonyl stretch (C=O).^34−37^ Alginate shows an absorption band at 1604.64, 1417.68 cm^–1^ due to symmetrical vibrations of the COO^–^ group, 1305.80 cm^–1^ due to *v* C–O str, and 1037.70 cm^–1^ in the saccharide region due to guluronic units of the *v* CO–C group. Also, the saccharide region aids in the formation of spherical structures.^38^ The polymer PHBV showed major absorption peaks at 3435.22, 2976.16, 2931.80, 2872.00, and 1722.43 cm^–1^ due to the C=O stretch of the ester group,^39^ 1454.32 cm^–1^ due to asymmetric deformation of a methylene group, and 1379.10 cm^–1^ due to the symmetrical wagging of the CH_3_ group, 1284.59 cm^–1^, peak at 1228.65 cm^–1^ (which is pure because of crystalline helical chains), 1186.22, 1134.14, 1101.35, and 1056.99 cm^–1^ due to antisymmetric −C–O–C– stretching, 979.83, 896.89, 623.00, 678.94, 516.92, 459.05, and 399.26 cm^–1^.^39^ CaCO_3_ showed major absorption peaks at 2364.02, 1504.38, 1086.13, 879.53, 744.46, and 664.52 cm^–1^ due to its crystalline behavior, which are prominent characteristics of the presence of carbonate ions. The peaks observed at 879.53 and 1086.13 cm^–1^ are due to symmetrical stretching and wagging of carbonate ions.^40^

It has been reported that Fourier transform infrared (FTIR) studies help to establish the intermolecular interaction between drugs and excipients.^25^ In our work, it has been noticed that the characteristic peaks of the drug were retained in the OF, indicating that the excipients did not alter the chemical properties of the drug. Thus, the FTIR analysis of the OF confirmed the compatibility of the drug with the excipients used in the colloidosomes. This shows that drugs have negligible or no interaction with the excipients. This was further supported by the similar results observed in the PM of the drug and excipients (1:1).

#### 3.3.2 XRD Studies

**Figure 5:**
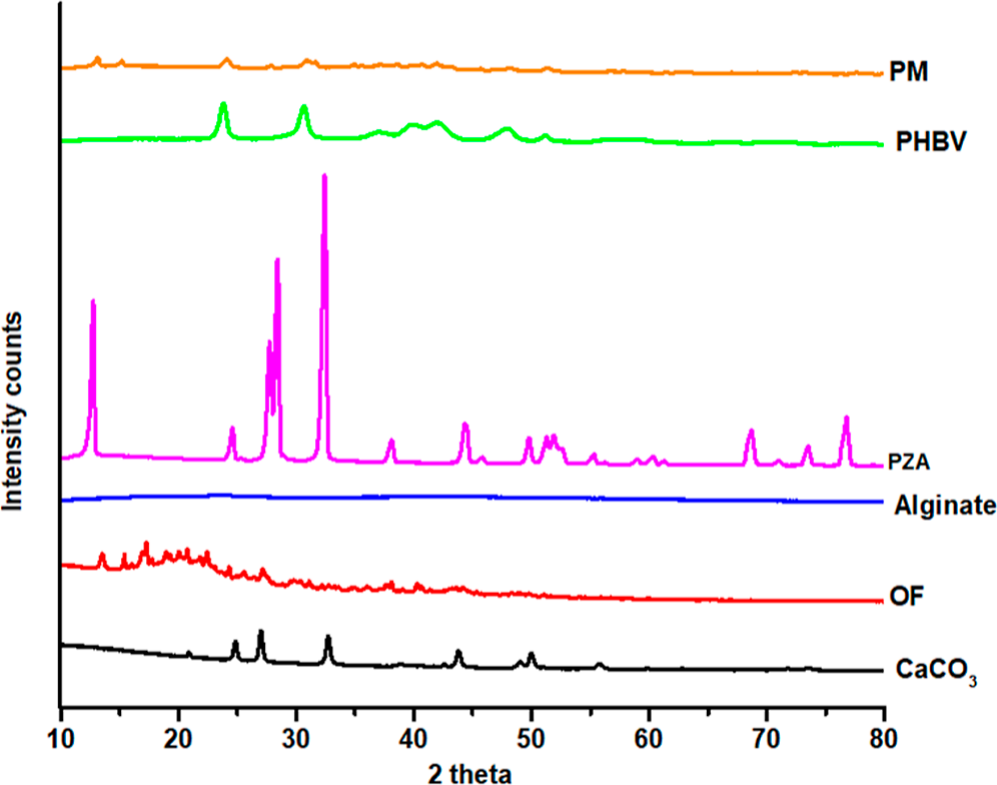
PXRD diffractogram of CaCO_3_, OF, alginate, PZA, PHBV, and PM (1:1).

The X-ray diffractograms of the drug (PZA), PM, OF, and other individual excipients (GDL, alginate, PHBV, and CaCO_3_) are shown in Figure 5.

PZA showed a typical XRD pattern with a characteristic peak at 2θ values of 7.89, 13.8, 15.37, 15.69, 17.7, 20.57, 23.72° and various other peaks in the range of 25–35°, respectively.^34^ Alginate showed a broad semicrystalline peak at 13.46° and another at 21.42° and exhibited an amorphous nature.^41,42^ CaCO_3_ shows sharp crystalline peaks at 20.86, 24.84, 27.02, 32.7, 38.84, 43.78, 50.0, 55.78, 59.78, 62.94, and 68.76°, most of which are peaks of vaterite.^27^ The polymer PHBV exhibits a very sharp peak at 13.4, 16.84, and 25.51°, respectively.^43−45^ The OF retains the peak of the drug and other excipients at 8.02, 9.06, 13.52, 15.4, 17.28, 18.94, 20.06, 21.82, 22.42, 24.3, 25.6, 27.14, 29.84, 38.12, 40.26, 43.6, and 44.22°; however, the intensity is reduced compared to a pure drug, which could be probably due to polymer coating and well-blended formulation.

#### 3.3.3 Differential Scanning Calorimetry

**Figure 6:**
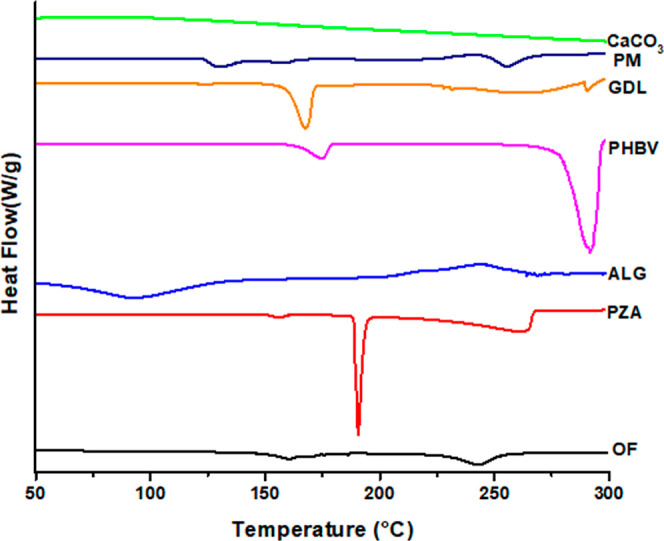
Thermograms of OF, PZA, alginate, PHBV, GDL, PM (1:1), and CaCO_3_.

The thermal analyses of the pure PZA, excipients used (GDL, alginate, PHBV, and CaCO_3_), PMs, and the OF were performed using DSC. The results showed that pure PZA had a sharp peak at 190.68 °C, indicating its melting point, crystalline nature, and purity (Figure 6).

The individual excipients, such as alginate, showed an endothermic peak at 93.83 °C, likely due to the elimination of water molecules, and a small exothermic peak at 243.79 °C.^39^ The PHBV polymer exhibited small endothermic peaks at 174.23 and 291.32 °C.^39^ GDL displayed distinct endothermic peaks at 167.57 °C and a broad endothermic peak at 261.70 °C. The PM also displayed endothermic peaks at 130.70, 158.48, and 256.28 °C and an exothermic peak at 241.82 °C. The DSC thermogram of the OF did not show any characteristic peaks of pure PZA, indicating the loss of crystallinity during thermal analysis due to various excipients used in the colloidosomes.

#### 3.3.4 Degradation Studies (TGA-DTA)

**Figure 7:**
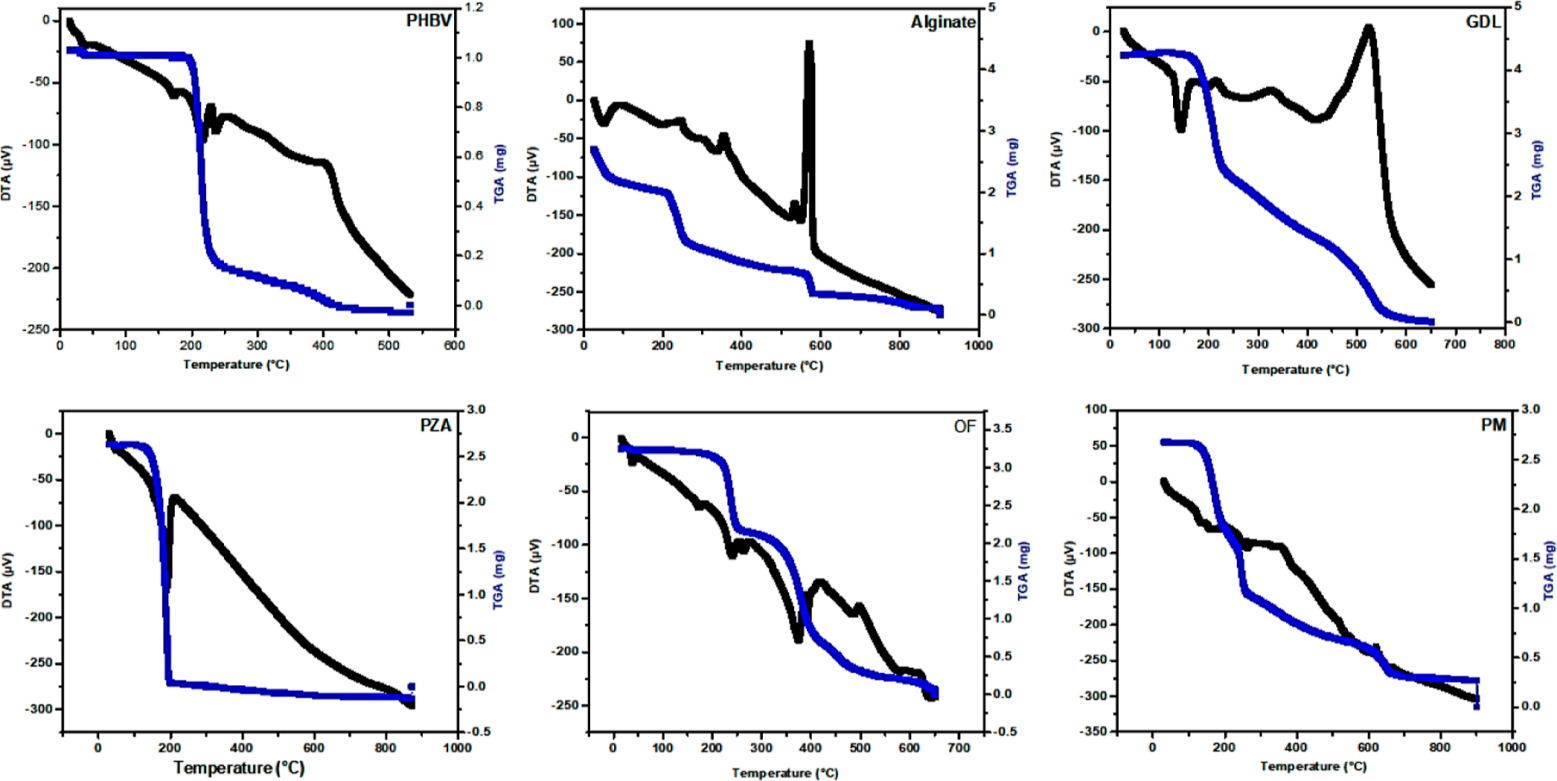
DTA-TG thermograms of PHBV, alginate, GDL, PZA, OF, and PM (1:1).

The mapping of endothermic and exothermic peaks is provided by DSC and DTA analysis.^46^ TGA also measures changes in thermal events by monitoring changes in sample mass as a function of temperature.^47^ The endothermic peak at 189.97 °C in the DTA curve corresponds to the melting point of the pure drug under inert atmospheric nitrogen (Figure 7).

The TGA of pure PZA showed a thermal decomposition of 98.09% with a weight loss between 134.77 and 204.09 °C at a single stage. This is consistent with the findings reported by Ngilirabanga *et al.*, 2020.^36^ The thermogram of sodium alginate showed a total weight loss of 95.55% in multiple steps, starting at 213 °C, followed by 259.27 and 565.5–582.7 °C due to the loss of water, cross-linking, and partial carbonization. Moreover, the DTA peaks also supported the TGA results.

The first stage was endothermic, requiring energy to evaporate adsorbed water molecules, while the other stages were exothermic, releasing energy due to burning or forming new chemical bonds.^48,49^ Similarly, GDL showed a weight loss of 40% between 165.17 and 227.49 °C, and the thermal analysis of the PM showed an initial weight loss of 55.05% in the range of 130.21–258.27 °C, followed by a second loss of 30.71% from 258.27 to 665.91 °C, resulting in a total loss of 89.81% until 902.24 °C. The OF showed signs of decomposition in the temperature range of 218.58–254.18 °C, as seen in the TGA curves, with a weight loss of 27.16%. Further disintegration was observed in the second stage of the TGA curve, with a faster decline in the temperature range of 254.18–650.47 °C and a weight loss of 65.43%, resulting in a total weight loss of 97.83%. The DTA curve of the OF showed no peak in the melting point region of the drug, indicating that the drug is in an amorphous state. This is supported by the DSC and PXRD data. Formulating PZA in this way enhanced thermal stability, as seen in the shift of the PZA’s larger endothermic peak to 376.04 °C.^50^

#### 3.3.5 FESEM Studies

**Figure 8:**
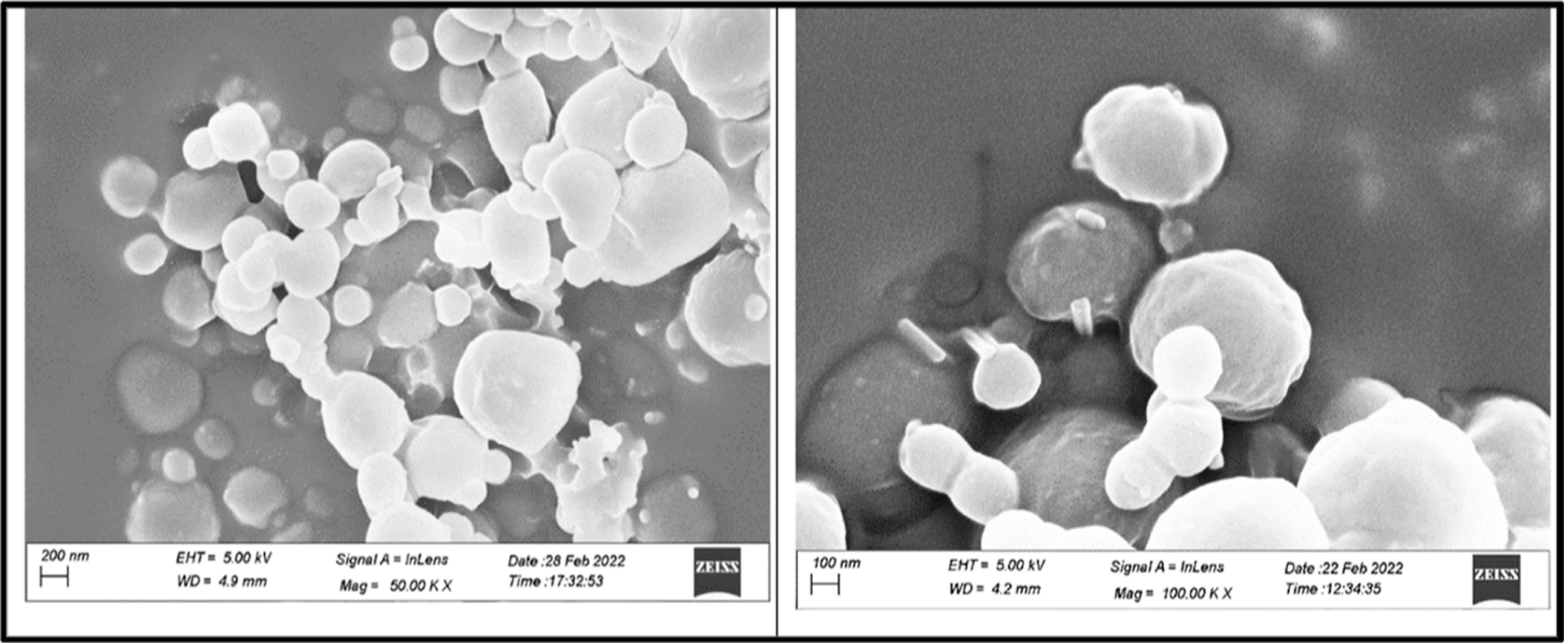
FESEM images of the OF at different magnifications.

The FESEM images of the OF (Figure 8) display spherical particles with a PHBV polymer coating at various magnifications. The OFs exhibited particle sizes ranging over 79–319 nm with a mean particle size of 165.6 nm. The spherical shape and porous coating of colloidosomes make them a desirable drug delivery system for further investigation.

### 3.4 Pharmacokinetic Analysis

**Figure 9:**
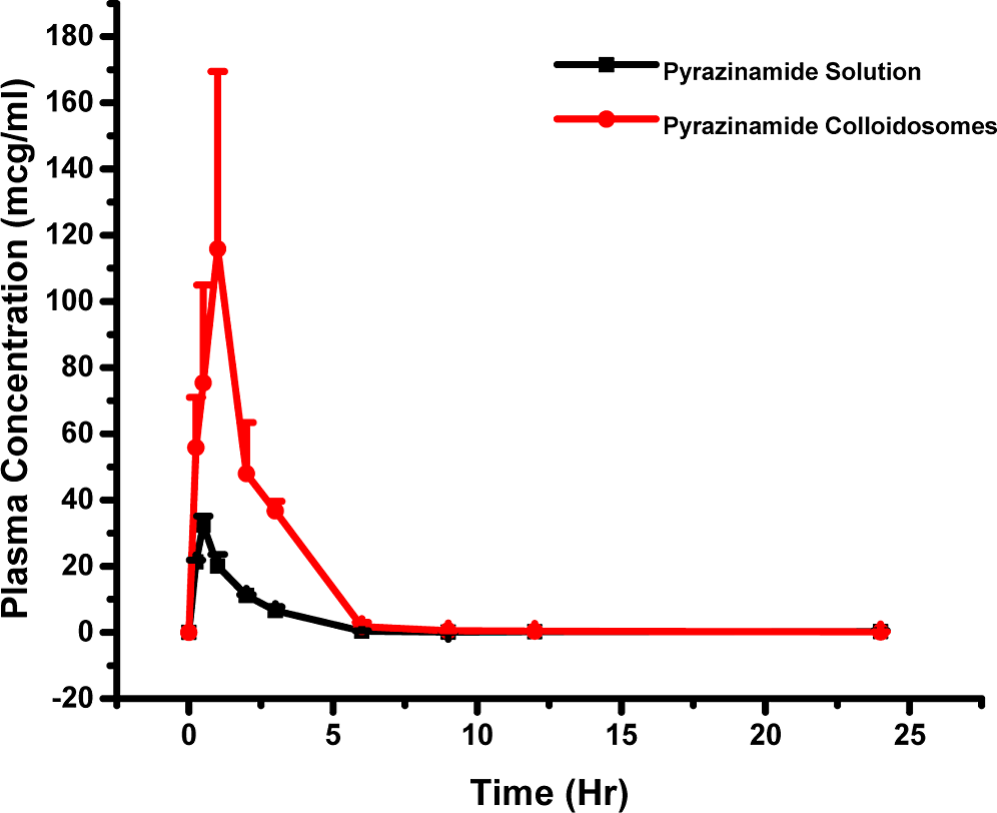
Pharmacokinetic studies plot the observed plasma concentration–time profile of the PZA solution and PZA-loaded colloidosome.

The plasma levels at different time points were evaluated by a validated LC–MS/MS method. The mean plasma concentration–time profiles for both PZA solution and PZA-loaded colloidosomes are illustrated in Figure 9. The PKPlus module of GastroPlus software was used to compute the pharmacokinetic parameters using non-compartmental and compartmental analysis methods.

The LC-ESI-MS/MS method in positive ionization mode was used to determine the concentration of pure PZA in the samples, and the method was validated. This mode provided improved sensitivity and selectivity for both PZA and the internal standard. The mass transition of Q1:Q3 *m*/*z*, 124.00/78.90 *m*/*z* for PZA and Q1:Q3 *m*/*z* 325.10/109.10 *m*/*z* for escitalopram, was monitored. The retention times for PZA and escitalopram were 2.2 and 2.6 min, respectively, with a total run time of 3 min. A linear calibration curve was created with a range of 0.2057–97.50 mcg/mL and plotted against the peak area ratio of PZA to escitalopram. The equation was *y* = 0.0325*x* + 0.0432, (*r*^2^ = 0.9911). The lowest limit of quantification for quality control was 0.2067 ng/mL for PZA. The plasma samples containing PZA and the internal standard (escitalopram) were analyzed after 30 mg/kg oral administration of PZA-loaded colloidosomes. The results showed no interference from endogenous plasma components. The accuracy of PZA within a day was 93.82–99.22%, with a precision of 0.03–0.80%. The accuracy between days was 94.62–99.31%, with a precision of 0.04–0.77%. These results meet the standards set by the U.S. Food and Drug Administration (2013). The average extraction efficiency of PZA was between 61.09 and 66.63%, with variability of 2.65–3.36%. The mean recovery of the standard escitalopram was 80.77%. The extraction recoveries were evaluated at three levels of quality control (QC), specifically 0.56, 36.25, and 72.50 ng/mL. The average matrix effect was 90.21 and 98.88% for LQC and HQC, respectively, indicating no significant matrix effects. The results of the freeze–thaw cycles for LQC and HQC quality control samples had an accuracy of 92.08 and 98.70%, respectively, while the accuracy of newly prepared LQC and HQC samples was 93.40 and 100.07%, respectively, showing acceptable freeze–thaw stability.

The oral pharmacokinetics of PZA in both colloidosomes and solution were evaluated using a validated LC–MS/MS method, which is a technique commonly used in the pharmaceutical industry for the quantification of drugs in biological samples. The mean concentration–time plots of the PZA solution and PZA-loaded colloidosomes are shown in Figure 9. These plots provide a visual representation of the drug concentration in the plasma over time and allow for the comparison of the pharmacokinetics of the drug in both forms. To further understand the pharmacokinetics of PZA in colloidosomes, the pharmacokinetic parameters were calculated using both non-compartmental and compartmental analysis with the GastroPlus software. Compartmental analysis is a method used to model the pharmacokinetics of drugs based on the assumption that the body can be divided into compartments, each with different rates of drug uptake and elimination. In this case, the best fit for PZA-loaded colloidosomes was determined to be two-compartment modeling based on the low Akaike’s Information Criterion (AIC) and Schwartz Criterion (SC) and high *r*^2^ values. The non-compartmental analysis was also conducted, and it gave the mean AUC_0–*t*_ of 61.24 and 260.9 mcg-h/mL and *m*ean *C*_max_ values of 32.386 ± 2.7444 and 115.868 ± 53.581 mcg/mL for PZA solution and PZA-loaded colloidosomes respectively. These values indicate that PZA-loaded colloidosomes demonstrated a 4.26-fold enhancement of oral absorption compared to the PZA solution. This suggests that the colloidosomes have the potential to be an effective drug carrier for delivering PZA to the target site in the body.

## 4 Conclusions

PZA-loaded colloidosomes were effectively prepared through the gelation method utilizing CaCO_3_, GDL, alginate, and PHBV. The influence of all excipients used in the formulation was examined. The utilization of Design Expert software and CCD facilitated the identification of the optimal formulation with minimal experimentation. Optimal levels of 150 mg of CaCO_3_, 2% sodium alginate, and 400 mg of PHBV were identified for the PZA-loaded colloidosome formulation, with an entrapment efficiency of ∼60%. The morphology of the particles and the effect of coating with the PHBV polymer were confirmed *via* the FESEM study. Drug and excipient interactions were evaluated using FTIR, XRD, DSC, and DTA-TGA, indicating the absence of incompatibility between the formed colloidosomes and the used excipients. GastroPlus simulated the plasma concentration profiles and revealed a 4.26-fold enhancement of oral absorption. The study shows that PZA-loaded colloidosomes have the potential as an oral delivery system for PZA.

## Data Availability

All data that support the findings of this study are included in the article.
